# Post-discharge health education for patients with enterostomy: A nationwide interventional study

**DOI:** 10.7189/jogh.13.04172

**Published:** 2023-12-13

**Authors:** Lu Zhou, Fengjiao Zhang, Hui Li, Ling Wang

**Affiliations:** 1 Department of Nursing, Peking University People's Hospital, Beijing, China; 2 School of Nursing, Peking University, Beijing, China

## Abstract

**Background:**

After discharge, patients with enterostomy face problems with poor self-nursing ability and low levels of psychological and social adjustment, which, without timely intervention, seriously affect their quality of life. We delivered health education to discharged enterostomy patients based on a WeChat health management program and evaluated its impact on their ostomy self-care ability and psychosocial adaptation level.

**Methods:**

Based on the WeChat health management program, we conducted continuous health education in the first, third, seventh, 11th, and 23rd weeks after discharge of enterostomy patients/before temporary enterostomy restoration to observe its impact on their self-care ability and psychosocial adaptation levels, as evaluated by an ostomy self-care ability questionnaire and ostomy adjustment inventory-20 checklist.

**Results:**

We included 4201 patients with enterostomy. Our findings showed that the self-care score of patients with enterostomy at discharge (baseline) (mean = 15.23, standard deviation (SD) = 5.22) was lower than that after intervention (mean = 17.71, SD = 1.28) (*P* < 0.05). The enterostomy psychosocial adaptation score of the enterostomy patients at discharge (baseline) (mean = 44.59, SD = 9.82) was lower than that after intervention (mean = 50.25, SD = 12.97) (*P* < 0.05).

**Conclusions:**

Health education for enterostomy patients after discharge can improve their self-care ability and psychological adaptation. Future studies could further explore the views and attitudes of this population toward health education based on the WeChat health management program.

Colorectal cancer has the third highest incidence and second highest mortality rate globally, with 1.5 million new cases and 525 000 deaths each year [1], with projections estimating more than 2.2 million new colorectal cancer cases and 1.1 million additional deaths would occur globally by 2030 [2]. As the number of cases of colorectal cancer continues to increase, so does the number of patients with enterostomy [3]. The concept of enhanced recovery is widely promoted in colorectal cancer patients, as it helps shorten the length of hospital stay [4,5] and likewise the time it takes for enterostomy patients to master ostomy-related skills.

Enterostomy patients are faced with physiological, psychological, social, and spiritual challenges after discharge [6], which can adversely impact their quality of life, psychosocial adaptation, and self-care [7-11] and even lead to anxiety and depression [12]. For example, several studies have shown that the level of psychosocial adaptation in this population is closely related to quality of life, self-efficacy, stigmatisation, and disability acceptance [11,13,14]. Moreover, enterostomy patients have varying needs between stages after discharge; for instance, they have a need of acquiring knowledge about enterostomy care, identifying related complications and receiving psychological support during the first three months post-discharge, and returning to the community three to six months post-discharge; consequently, meeting these needs is a prerequisite for improving their quality of life [15]. In turn, post-discharge health education could be the possible answer to this challenge.

Health education is a continuous, dynamic, complex, and planned teaching-learning process delivered throughout a lifespan and in different settings, and implemented through an equitable and negotiated client and health professional “partnership” to facilitate and empower the person to promote/initiate lifestyle-related behavioral changes that promote positive health status outcomes [16]. Health education is a prerequisite for patients to improve their self-management skills and disease control [17]. WeChat, an instant messaging app for smart devices, is widely used in China to promote health and self-management, such as transition care for patients with chronic diseases [18], post-operative patients [19].

Current studies of enterostomy patients have focused on the period of hospitalisation, and only a few recognized differences in their needs at different stages (after discharge) [15]. Moreover, enterostomy patients are willing to improve their self-care ability by learning ostomy-related knowledge [20], providing a possibility for delivering health education. To further understand the impact of health education based on WeChat health management program on the psychosocial level and self-ostomy care ability of enterostomy patients after discharge, we conducted a study in 202 hospitals across the country.

## METHODS

### Study design

We set up a national quasi-experimental study investigating the effects of health education on psychosocial adaptation and self-care ability in enterstomy patients.

### Settings and participants

We selected 202 hospitals in 30 provinces and administrative districts as the survey points from July 2021 to July 2022 using convenient sampling method. We included patients aged ≥18 years undergoing ostomy surgery, with a tumor that had no distant metastasis; likewise, the person or their caregiver had a smartphone, and they had to voluntarily participate in this research and provide informed consent. We excluded people with visual and hearing impairment (and other conditions) resulting in difficulty in communication and reading mobile phone information; unable to use mobile phone feedback information; with experience in ostomy nursing; suffering from serious illness, physical activity disorder, or inability to take care of himself/herself; with other types of cancer.

### Data collection and measures

An education nurse managing the patient collected data before discharge (baseline) and 23 weeks after discharge/before temporary ostomy restoration. After encryption, all data was dynamically transmitted and securely stored in the server of health management program to ensure the accuracy and standardisation; simultaneously, the system monitored data quality in real time.

### Ostomy self-care ability questionnaire

We used the Self-care Ability Questionnaire for Enterostomy compiled by Taiwan scholar Gao Qiwen. The questionnaire aims to understand whether patients had the knowledge of self-observation of enterostomy, perioral skin care, dietary principles, odour control, activity principles, etc. It comprises 21 questions, with an overall score range from 0 to 21 points. We considered a total score ≥13 as a higher score and <13 as a lower score; the higher the score, the better the mastery of self-care knowledge and skills. Cronbach's α of this part of the scale was 0.82 [21].

### Ostomy adjustment inventory-20 (OAI-20)

The English version of Ostomy adjustment inventory-23 (OAI-23), developed by Simmons et al. in 2009, consists of 23 items, each measured on a 5-point Likert scale (0-4), with higher scores indicating better adaptive ability [22]. In 2010, Xu et al. [23] translated the OAI-23 scale into Chinese and produced the OAI-20 scale. The total score of the OAI-20 ranges from 0 to 80, with higher scores indicating higher levels of psychosocial adaptation (low: <40, moderate: 40-59, high: ≥60). The scale is divided into three dimensions: positive emotion, negative emotion, and social life adaptation, in which negative emotion adopts a reverse scoring method. Cronbach's α coefficient of this scale is 0.8686, and the reliability retest is 0.68.

### Health education checklist

Our team developed an educational checklist for enterostomy patients after three rounds of meetings with Ostomy Expert Group members, with each meeting consisting of ≥10 members. This process led to the development of a 12-item health education list on the daily care of enterostomy patients, the care of enterostomy and surrounding skin, the use and replacement of enterostomy bags, psychological and nutritional guidance, etc. The answers to the question are set to “clear”, “partially unclear”, and “completely unclear”/“yes”, “no”. We applied the checklist for the first health education and the health education guidance of patients after discharge **(**Table S1 in the **Online Supplementary Document**).

### Content of health education

#### Expert team

We set up an expert team to standardise the project implementation process, form a health education checklist, provide advice for the construction of the health management program, and guide the smooth implementation of the project. Expert team members included clinical ostomy care specialists, ostomy education specialists, and care administrators. To be included in the team, they had to have had a bachelor's degree or above; intermediate professional and technical titles or above; more than five years of experience in ostomy treatment/nursing, ostomy nursing education, or ostomy nursing management. They experts identified the possible needs of enterostomy patients at different stages of post-discharge and the program of health education before the plan was implemented.

#### Education nurses team

The education nurses were mainly responsible for selecting patients, implementing health education after discharge, and collecting data. They were recruited by the study hospitals (at least one education nurse was recruited) and had to be engaged in enterostomy care and with more than five years of experience in enterostomy care. We organized training for the education nurses before the study started, explaining the purpose and significance of the study and detailing the implementation process. Only those nurses who passed the training and assessment were allowed to participate in the study.

#### Technical support

We designed and developed the health management program in September 2020 with support from the Beijing Great Commonwealth Foundation and an information technology company. The program included a patient side and a nurse side (Figure S1-S2 in the **Online Supplementary Document**). The main functions of the patient side of program mainly included follow-up appointment, photo diagnosis and health consultation, participation in “Patient Church” activities (including knowledge answers, sharing of patient ostomy nursing experience, etc.), and access to health education knowledge related to ostomy nursing.

### Research and implementation process

#### First health education

On a day before and at discharge, the health education nurse introduced the contents and specific procedures of the program (including the purpose, significance, method, possible benefits, and risks) to the eligible enterostomy patients, after which they voluntarily enrolled and signed the informed consent. This introduction lasted about 10 minutes; the education nurses tried to ensure that patients truly understood the process, informed patients of the principles of confidentiality, voluntariness and withdrawal at any time, and assisted patients to log in to the health management program for the first time after patients they agreed to participate and provided informed consent. Discharge guidance was completed according to the health education checklist and education manual. Meanwhile, education nurses guided patients in completing a baseline survey, which included the patients’ basic information (region, type of enterostomy, ethnic group, religion, education level, self-care ability, primary caregiver, residency, medical payments method), as well as the ostomy self-care ability questionnaire and OAI-20.

#### Post-discharge health education

After expert discussion, we determined that the health education for patients with enterostomy should be implemented in the first, third, seventh, 11th, and 23rd after discharge through the health management procedure. The educational content was based on the health education checklist for ostomy patients and focused on resolving uncertainties on the problems they faced. The health education mainly included the following content: paying attention to patients’ views on enterostomy and enterostomy complications, emphasizing the importance of enterostomy and skin care around enterostomy, arousing patients’ attention to enterostomy care, and promoting their active self-care of enterostomy. In this way, educational nurses focused on the major health problems recently encountered by patients and attempting to provide solutions. Throughout the study period, the patients were able to consult with the mini-program by leaving messages and taking photos, and health education nurses answered online or by phone. The study ended at week 23 after discharge or before temporary enterostomy closure, at which time enterostomy patients filled in the ostomy self-care ability and OAI-20 questionnaires.

### Statistical analysis

We analyzed data using SPSS, version 26.0 (IBM, New York, NY, USA), presenting patients’ demographic disease characteristics with frequencies, percentages, and cumulative percentages and their self-care ability and psychosocial adaptation score with means with standard deviations (SDs). We used the Kolmogorov-Smirnov test to check the normality of data distribution; in cases of non-normality, we used the Box-Cox transform to convert it to a normal distribution. We used the paired sample *t*-test to assess the difference in continuity variables between the two groups. All statistical analyses were bilateral, with a *P*-value <0.05 denoting statistical significance.

### Ethical considerations

The Peking University People's Hospital (2021PHB172-001) approved the protocol. The privacy of participants was protected by non-identifiable information. All participants signed an electronic written informed consent form; participation was voluntary and they could have withdrawn at any time.

## RESULTS

### Sample description and demographic disease characteristics

We included 4483 eligible enterostomy patients; 282 (6.35%) were lost to follow-up, meaning 4201 completed the study (Figure S3 in the **Online Supplementary Document**).

Of the total sample, 63.96% were patients with temporary enterostomy, 36.04% were patients with permanent enterostomy, 57.72% were junior high school (and below), and 24.47% were high school students. Overall, 30.2% of enterostomy patients had complete self-care, while 58.13% were partially and 11.85% completely dependent on others for care (**Table 1**).

**Table 1:** Demographic disease characteristics of the study population

Variables	n (%)	Cumulative percentage
Region		
*East*	2334 (55.56)	55.56
*West*	1050 (24.99)	80.55
*Middle part*	817 (19.45)	100.00
Type of enterostomy		
*Temporary*	2687 (63.96)	63.96
*Permanent*	1514 (36.04)	100.00
Ethnic group		
*Han nationality*	4070 (96.88)	96.88
*Hui nationality*	19 (0.45)	97.33
*Manchu*	15 (0.36)	97.69
*Tujia nationality*	14 (0.33)	98.02
*Other ethnic minorities*	83 (1.98)	100.00
Religion		
*No religion*	4180 (99.50)	99.50
*Christian*	8 (0.19)	99.69
*Buddhism*	8 (0.19)	99.88
*Islam*	5 (0.12)	100.00
Education level		
*Junior high school (and below)*	2425 (57.72)	57.72
*High school*	1028 (24.47)	82.19
*College*	417 (9.93)	92.12
*Undergraduate*	298 (7.09)	99.21
*Master*	24 (0.57)	99.78
*Doctor*	9 (0.22)	100.00
Self-care ability		
*Completely self-care*	1261 (30.02)	30.02
*Partially self-care*	2442 (58.13)	88.15
*Completely dependent*	498 (11.85)	100.00
Primary caregiver		
*Children*	1892 (45.04)	45.04
*Spouse*	1657 (39.44)	84.48
*Their own*	396 (9.43)	93.91
*Other*	148 (3.52)	97.43
*Parents*	108 (2.57)	100.00
Residency		
*Town*	2507 (59.68)	59.68
*Countryside*	1694 (40.32)	100.00
Medical payments		
*Health insurance*	3832 (91.22)	91.22
*Completely at their own expense*	261 (6.21)	97.43
*Publicly-funded medical care*	108 (2.57)	100.00
Total	4201 (100.00)	100.00

### Ostomy self-care ability

The data showed that the self-care ability scores of enterostomy patients (at the 23^rd^ week after discharge or before temporary enterostomy closure) (mean = 17.71, SD = 1.28) (*P* < 0.001) was higher than at discharge (baseline) (mean = 15.23, SD = 5.22) (**Table 2**).

**Table 2:** Analysis of psychosocial adaptation and self-efficacy in ostomy patients before and after intervention*

Outcomes	Before intervention, mean (SD)	After intervention, mean (SD)	T-value	*P*-value
Score for ostomy self-care ability (n = 4201)	15.23 (5.22)	17.71 (1.28)	-31.20	<0.001
Psychosocial adaptation score (n = 4201)	44.59 (9.82)	50.25 (12.97)	-25.38	
Psychosocial adaptation				
*Positive emotions*	13.70 (3.50)	14.94 (3.15)	-18.74	
*Negative emotions*	16.68 (7.48)	19.76 (8.77)	-19.08	
*Social life adaptation*	14.23 (2.98)	15.75 (3.37)	-23.34	

SD – standard deviation

*All outcome indicators were the Box-Cox transform (they did not conform to normal distribution by normality test, Kolmogorov-Smirnov test: *P* < 0.05).

### Ostomy psychosocial adaptation

We found that the patients’ psychosocial adaptation score at discharge (baseline) (mean = 44.59, SD = 9.82) was lower than after the intervention (at the 23rd week after discharge or before temporary enterostomy reclosed) (mean = 50.25, SD = 12.97) (*P* < 0.001). The scores of positive emotion (mean = 14.94, SD = 3.15), negative emotion (mean = 19.76, SD = 8.77), and social life adaptation (mean = 15.75, SD = 3.37) were significantly higher after than before the intervention (positive emotion: mean = 13.70, SD = 3.50; negative emotion: mean = 16.68, SD = 7.48; social life adaptation: mean = 14.23, SD = 2.98) (*P* < 0.001).

## DISCUSSION

Our findings suggest that post-discharge health education based on health management program is effective in improving patients' ostomy self-care ability and psychosocial adaptation, as they had significantly higher scores after receiving health education compared to baseline.

Enterostomy patients’ needs after discharge and their related problems were resolved through the WeChat-based health management program, as it met their needs of regarding enterostomy-related knowledge and improved their in nursing enterostomy. We thus aim to hold further offline or online “patience church” activities through which they can share enterostomy nursing skills, which could further stimulate them to actively learn ostomy-related skills and improve their enterostomy nursing ability and psychosocial adaptation level [24]. Previous studies [25] have also found that group interaction can help improve the psychosocial adaptation level of enterostomy patients, while several have found health education to be positive for this population. For example, Ko et al. [26] found that multimedia education for enterostomy patients helped improve their self-care ability and quality of life, while Wang et al. [27] also found that the application of home care mobile app can improve the psychosocial adaptation level of enterostomy patients after discharge, which is helpful in improving their psychosocial adaptation level. However, we found that previous studies on health education for patients with enterostomies had certain shortcomings; for example, the design of their health education content did consider the changing needs of the patients, the sample size was insufficient and was conducted in a single center, and many had high rates of loss to follow-up at the end of health education [28-30]. Our study addressed all said shortcomings.

Enterostomy is different from other surgical procedures in that it is not closed immediately after surgery, but will stay with the patient for a period of time or permanently [31]. Enterostomy patients face a variety of problems in the postoperative period and their needs change over time; for instance, in the early stages of enterostomies, the patients have a high need to acquire knowledge of enterostomy care, which will affect the level of psychosocial adaptation if their needs are not met [15,32,33]. Therefore, it is important to design a health education program that is scientific, rational, and responsive to the enterostomy patients’ needs. We designed such a program here, which comprised a standardised post-discharge health education led by nurses, the contents of which were discussed and decided by enterostomy experts, ensuring scientific and rational content. Previous studies have found that enterostomy patients, under the guidance of enterostomy specialist nurses, have a more comprehensive grasp of relevant knowledge and a higher degree of acceptance of knowledge, which is conducive to improving their ability of enterostomy nursing and the level of psychosocial adaptation [25,34]. Moreover, the enterostomy experts jointly formulated the follow-up time according to the needs of enterostomy patients. The first follow-up time was the one week after discharge (to consolidate the knowledge related to enterostomy care), the second three weeks after discharge (to remove the support rod), the third seven weeks after discharge (the patient was ready to resume normal life), the fourth 11 weeks after discharge (the patient was ready to close the temporary enterostomy), and the fifth 23 weeks after discharge or before the temporary enterostomy closed (basic adaptation to the enterostomy life/knowledge after the enterostomy return). These five follow-up times are the time when enterostomy patients face the most problems, meaning personalised intervention for enterostomy patients based on “timing” are more helpful in meeting their needs.

Moreover, this study is based on WeChat applet for patients' health education, which is convenient for them to operate on their mobile phones and acquire the knowledge. If they cannot solve the problem, they can also leave a message or consult the health education nurse for professional guidance, which greatly facilitates the patients. Furthermore, this was a nationwide intervention study involving multiple health care institutions; thus, the positive effect of our health education program is likely meaningful for enterostomy patients. Finally, one of the important indicators to assess the feasibility of our health education program is the dropout rate; if it is high, it means that the program may not be suitable for the patients. We found that the dropout rate of the previous health education program for the patients with intestinal stoma was high, such as with the study of Yiğitoğlu et al. [30], where the dropout rate of the patients with intestinal stoma at the end of the health education was 26%. The dropout rate at the end of our study was 6.35%, indicating that our health education method was easy for patients to accept, and enterostomy patients benefited from it.

However, our study also has some limitations. We did not explore whether health education based on the WeChat health management applet had a more profound impact on patients with permanent enterostomies, and we also did nod investigate their feelings and feedback during the health education process. We also only measured the patients' self-care ability and psychosocial level at the beginning of the study and at the end of the follow up, and we did not learn about the longitudinal changes in their self-care ability and psychosocial level during the process.

## CONCLUSIONS

Health education for discharged enterostomy patients based on WeChat health management program can help improve their self-care ability and psychosocial adaptation level. Future studies could further explore the long-term effects of health education on patients with permanent enterostomy.

## Additional material


Online Supplementary Document
