# Supplementary material for: Post-discharge health education for patients with enterostomy: A nationwide interventional study
Source: J Glob Health. 2023 Dec 13;13:04172. doi: 10.7189/jogh.13.04172 (PMC10716631; doi:10.7189/jogh.13.04172)

Online Supplementary Document
Supplementary Tables/Figures

Table S1: Health Education Checklist

Item	Answers		
#1: Everyday diet, dress, bathing, exercise, socialising and work	Clear	Partially unclear	Completely unclear
#2: Prevention and management of stoma complications	Clear	Partially unclear	Completely unclear
#3: Prevention and management of peristomal skin complications	Clear	Partially unclear	Completely unclear
#4: Observation of excretions	Clear	Partially unclear	Completely unclear
#5: Discharge and replacement of ostomy bags	Clear	Partially unclear	Completely unclear
#6: Ways to reduce leakage from ostomy bags	Clear	Partially unclear	Completely unclear
#7: Frequency of replacement of ostomy bags used	Clear	Partially unclear	Completely unclear
#8: Types of ostomy bags, selection and use	Clear	Partially unclear	Completely unclear
#9: Types, selection and use of supplies related to ostomy care	Clear	Partially unclear	Completely unclear
#10: Access to ostomy Care Supplies	Clear	Partially unclear	Completely unclear
#11: Need for psychological counselling	Yes	No	
#12: Need for nutritional guidance	Yes	No	

Figure S1: Based on the wechat health management program: patient side

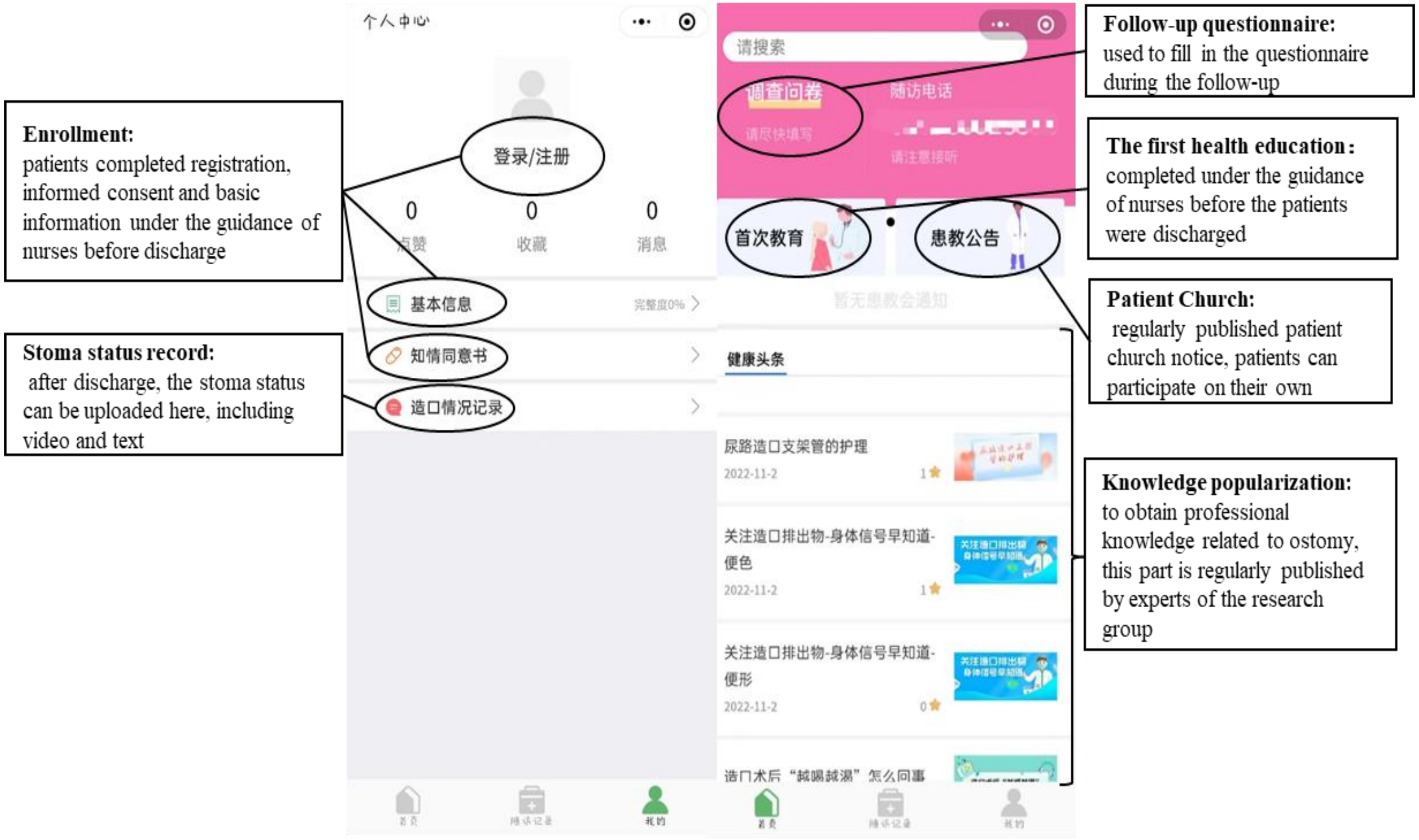

Figure S2: Based on the wechat health management program: nurse side

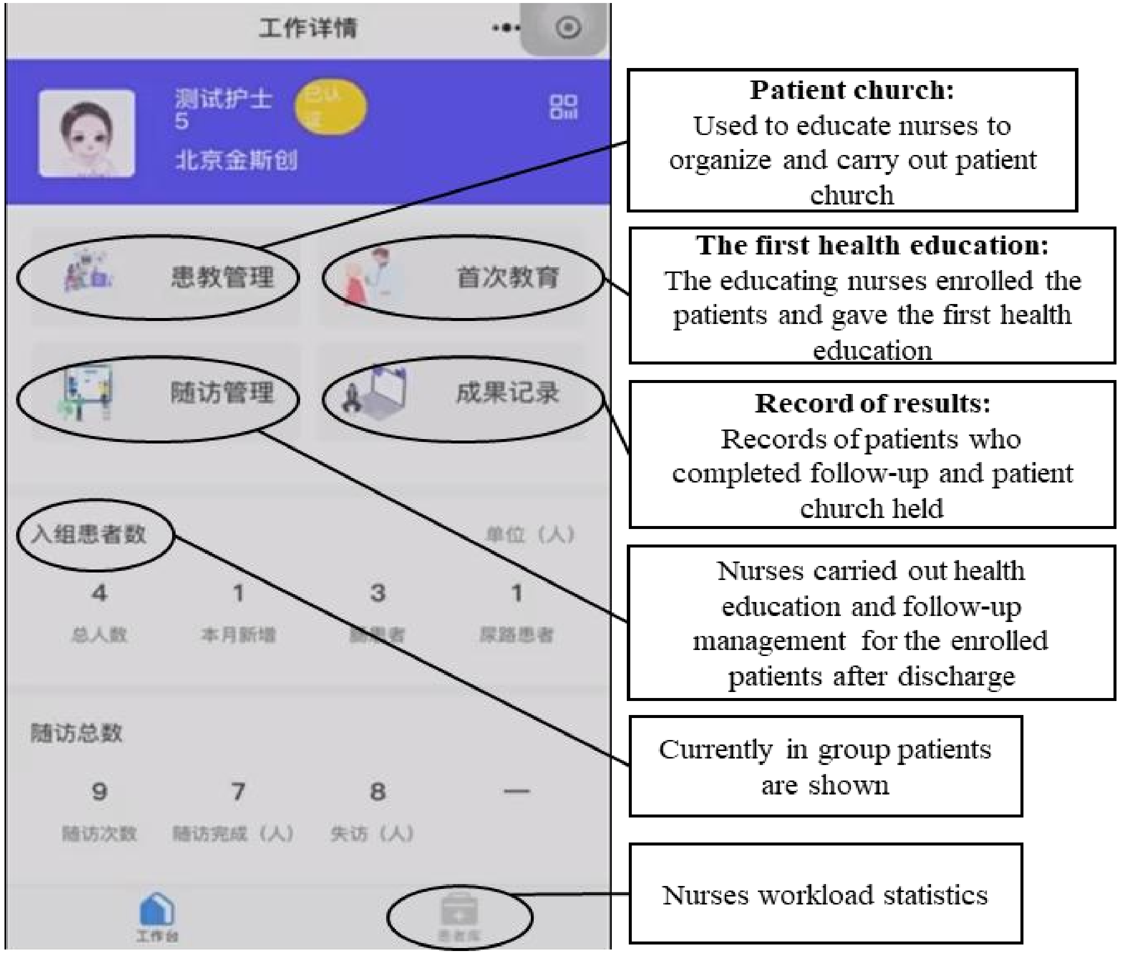

Figure S3: The participant screening process

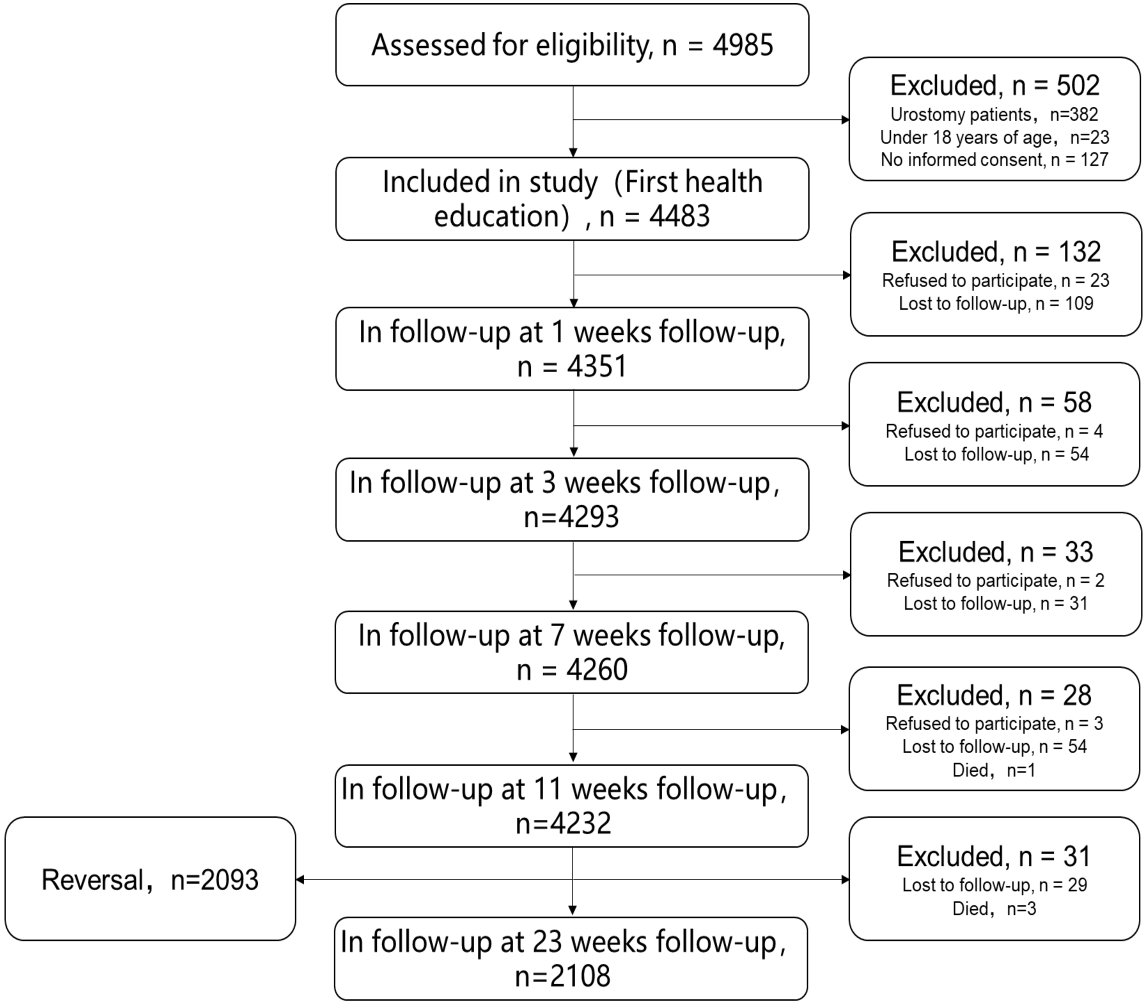

Supplement: Online Supplementary Document [file jogh-13-04172-s001.pdf]
